# Editorial: Neural plasticity for rich and uncertain robotic information streams

**DOI:** 10.3389/fnbot.2015.00012

**Published:** 2015-10-27

**Authors:** Andrea Soltoggio, Frank van der Velde

**Affiliations:** ^1^Computer Science, Loughborough UniversityLoughborough, UK; ^2^CTIT, CPE-BMS, University of TwenteEnschede, Netherlands

**Keywords:** neural plasticity, neural adaptation, neuro-robotics, cognitive modeling, emobodied cognition

Models of adaptation and neural plasticity are often demonstrated in robotic scenarios with heavily pre-processed and regulated information streams to provide learning algorithms with appropriate, well timed, and meaningful data to match the assumptions of learning rules. On the contrary, natural scenarios are often rich of raw, asynchronous, overlapping and uncertain inputs and outputs whose relationships and meaning are progressively acquired, disambiguated, and used for further learning. Therefore, recent research efforts focus on neural embodied systems that rely less on well timed and pre-processed inputs, but rather extract autonomously relationships and features in time and space. The bio-inspired focus does not seek the most effective machine learning method to solve those problems, it rather points toward a better understanding of problem solving mechanisms in neural systems, which can in turn also provide viable solutions to difficult problems.

Realistic models of plasticity must account for delayed rewards (Soltoggio et al., 2013a), noisy and ambiguous data (Soltoggio et al., 2013b), and emerging and novel input features during online and value learning (Krichmar and Röhrbein, 2013). Those factors have indeed been an emerging focus of search (e.g., Sporns and Alexander, 2003; Lungarella and Sporns, 2006; Martius et al., 2013), with a growing number of studies that cannot be reviewed in this short editorial. Such approaches model the progressive acquisition of knowledge by neural systems through experience in environments that may be affected by ambiguities, uncertain signals, delays, or novel features (Pugh et al., 2014; Soltoggio, 2015). This Research Topic in Frontiers in Neurorobotics explored fundamental properties and dynamics of neural learning systems that are naturally immersed in a rich information flow. We are pleased with the contributions collected in this Research Topic, each of which addresses key topics in this emerging and important field of research.

One overarching problem in this field is that of making sense of large amounts of data from sensory systems in order to recognize particular situations and perform basic tasks. Parisi and colleagues took a self-organizing neural approach to action recognition using human pose-motion features. The Growing When Required (GWR) networks manifest a high-level structural plasticity that regulates network complexity in relation to the task (Parisi et al., 2015). Such a bio-inspired approach recorded state-of-the-art performance on a dataset of full-body actions captured with a depth sensor, with competitive results in a public benchmark of domestic daily actions.

Another source of large, noisy and uncertain data is found in robotic tactile sensors. Chou et al. (2015) deployed a specific robot called CARL-SJR with a full-body tactile sensory area. CARL-SJR encourages people to communicate with it through gentle touch, and provides feedback to users by displaying bright colors on its surface. The time-delayed and uncertain nature of the interactions poses challenges to the formation of correct associations between stimuli, rewards and actions. The approach devised by Chou et al. (2015) experiments with a strongly bio-inspired architecture of spiking neurons with neuromodulated plasticity. The model abstracts brain areas such as the primary somatosensory cortex, prefrontal cortex, striatum, and the insular cortex to process noisy data generated directly from CARL-SJR's tactile sensory area. The result is a robust learning mechanism that reliably forms correct associations and preferences for directions without heavily pre-processed inputs.

Uncertainty and large amount of data are also found in collaborative multi-robot scenarios in which multiple robots work alongside humans. Galbraith and colleagues propose a motor babbling approach to learn a complex set of relations and interactions with the 11-degrees-of-freedom RoPro Calliope mobile robot (Galbraith et al., 2015). Motor babbling of its wheels and arm enabled the Calliope to learn how to relate visual and proprioceptive information to achieve hand-eye-body coordination.

Motor control is a problem in which neural plasticity results in high level of adaptation, adjusting neural systems to operate in combination with specific bio-mechanical structures and morphologies. (Burms et al., 2015) demonstrated the utility of modulated Hebbian plasticity in embodied computation for compliant robotics. In such scenarios, control policies are generally unknown due to the partial offload of control policies to morphological computation. Modulated Hebbian plasticity was shown to lead to hybrid controllers that naturally integrate the computations that are performed by the robot's body into a neural network architecture. Those results demonstrate the potential of universal applicability of plasticity rules to complex control problems.

A similar problem was tackled in Dasgupta et al. (2015) in which they used distributed recurrent neural networks with synaptic adaptation to find a range of complex behaviors for walking robots. In particular, their approach demonstrated a remarkable flexibility in designing control systems that can work with multi-legged robots. A Central Pattern Generator is used to feed a self-adaptive reservoir network, which in turn provides motor control through a read-out integration unit. These results contribute to demonstrate the efficacy and continuous advancement of plastic neural models in complex input-output control scenarios.

The overall vision provided by these research papers outlines an increasingly more effective deployment of plastic neural models to tackle complex perception and control problems in which noise, uncertainty and delays pose a challenge to many algorithms. This vision matches the intuition of bioinspired neurorobotics approaches that propose advanced, plastic neural systems as viable models when sensory-motor information flows approach the richness and complexity found in the behavior of biological systems. We foresee a continuous growing attention to this emerging research area, in particular related to the development of more effective, scalable and general neural learning algorithms to effectively tackle rich and uncertain robotic information streams.

## Author contributions

AS devised the structure. Both authors AS and FV formulated the content and wrote the paper.

### Conflict of interest statement

The authors declare that the research was conducted in the absence of any commercial or financial relationships that could be construed as a potential conflict of interest.
